# Development of Novel Silica-Based Formulation of α-Lipoic Acid: Evaluation of Photo and Thermal Stability of the Encapsulated Drug

**DOI:** 10.3390/pharmaceutics12030228

**Published:** 2020-03-04

**Authors:** Ekaterina S. Dolinina, Elizaveta Yu. Akimsheva, Elena V. Parfenyuk

**Affiliations:** G. A. Krestov Institute of Solution Chemistry of Russian Academy of Sciences, 153045 Ivanovo, Russia; akimsheva.98@mail.ru (E.Y.A.); evp@isc-ras.ru (E.V.P.)

**Keywords:** α-lipoic acid, silica, drug formulation, drug-silica interaction, photodegradation, thermal degradation, kinetics

## Abstract

Powerful antioxidant α-lipoic acid (LA) is easily degraded under light and heating. This creates difficulties in its manufacture, storage and reduces efficiency and safety of the drug. The purpose of this work was to synthesize novel silica-based composites of LA and evaluate their ability to increase photo and thermal stability of the drug. It was assumed that the drug stabilization can be achieved due to LA-silica interactions. Therefore, the composites of LA with unmodified and organomodified silica matrixes were synthesized by sol-gel method at the synthesis pH below or above the pKa of the drug. The effects of silica matrix modification and the synthesis pH on the LA-silica interactions and kinetics of photo and thermal degradation of LA in the composites were studied. The nature of the interactions was revealed by FTIR spectroscopy. It was found that the rate of thermal degradation of the drug in the composites was significantly lower compared to free LA and mainly determined by the LA-silica interactions. However, photodegradation of LA in the composites under UV irradiation was either close to that for free drug or significantly more rapid. It was shown that kinetics of photodegradation was independent of the interactions and likely determined by physical properties of surface of the composite particles (porosity and reflectivity). The most promising composites for further development of novel silica-based formulations were identified.

## 1. Introduction

In present time, a significant scientific and technological interest has focused on drug-silica materials, which have been identified as potentially effective drug delivery systems (DDS). Encapsulation of drugs in silica matrix and formation of composite material between drug and colloid silica can cardinally change some very important properties of the drug, e.g., enhance its solubility and dissolution rate [1,2], protect against decomposition under various environmental factors [3,4,5], modify release kinetics of the drug [6,7,8,9], etc. Thus, the DDS can improve the administration and efficacy of pharmaceutical compounds.

**Figure 1:**
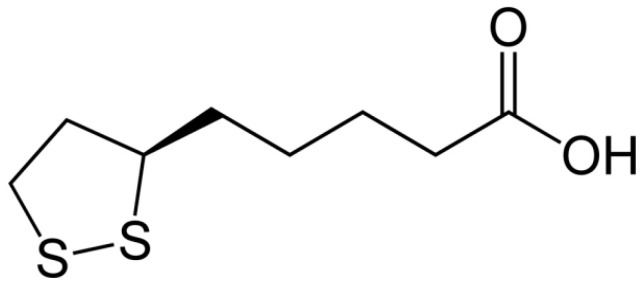
Structural formula of α-lipoic acid.

α-lipoic acid (thioctic acid) (LA) (Figure 1) is a powerful antioxidant widely used for treatment of various chronic diseases [10,11] and as anti-aging agent in cosmetology [12,13] but has a series of shortcomings that create difficulties in its manufacture, storage and significantly reduce its pharmacological efficiency.

In particular, LA is easily decomposed under light [14,15,16,17] and has a low thermal stability [18,19,20,21].

The works reported in literature have shown that encapsulation of drugs in silica materials can significantly increase their stability against degradation under light and heating. For instance, a series of works of Berlier et al. [4,22,23] showed that adsorption of flavonoids on mesoporous silica materials promotes enhancement of stability of the drugs against UV degradation. It was shown that the protective effect of aminopropyl functionalized silica is greater in comparison with bare silica due to a stronger interaction of quercetin aglycone with aminopropyl groups of NH2-MCM-41 [4]. Ruiz-Rico et al. found the protective action of the mesoporous silica against photodegradation and heating of folic acid in fruit juice [5]. Zhang et al. [24] reported that encapsulation of protease in silica matrix significantly increased thermal stability of the enzyme.

Therefore, it was assumed that encapsulation of LA in silica materials and formation of silica-based composite can lead to preparation of novel oral formulation of the drug with improved physicochemical and functional properties. It should be noted that no works devoted to development of silica-based composites of LA were found in literature. Only “soft” materials, that are deformed or structurally altered by thermal or mechanical stress and subject to enzymatic degradation, such as cyclodextrins, polysaccharides and lipids have been proposed to enhance thermal stability [25,26,27] and photostability [26,27,28] of the drug. Colloid silica is very promising material for development of oral pharmaceutical formulations, because it is recognized as a safe food additive [29]. Besides, in contrast to “soft” materials, silica has mechanical and thermal strength, as well as stability against enzymatic degradation and microbe attacks.

Thermo and photoprotection of the encapsulated drug can be achieved by creation of protective shell around the drug. For example, Jang et al. [30] showed that photostabilization of amlodipine loaded in dry emulsions was achieved due to photoprotective lipid layer around the encapsulated drug. The same conclusion was made by Raza et al. [31] when they investigated photostability of tretinoin encapsulated in solid lipid nanoparticles. Another mechanism of drug stabilization is its interaction with capsule material. The interactions of drugs with mesoporous silica [4], cyclodextrins [32], liposomal phosphatidylcholine [33] and polysaccharide microparticles [34] led to their increasing photo stability. Thermal stability of drugs was enhanced by complex formation with β-cyclodexrin [26,35] and amylose [26].

It is well known that modification of silica matrixes with various functional groups influences drug-silica interactions. The modification results in altering properties of silica matrixes, e.g., hydrophilicity/hydrophobicity, acid-base properties and surface charge; i.e., their surface reactivity. Therefore, the modification of silica matrix with appropriate functional groups can lead to strengthening drug-silica interactions and changes in the photo and thermal stability of drug in its composites with silica. Another factor influencing interactions in the composites is synthesis pH. Drugs are often weak acids or bases, and their ionization state depends on medium pH. In general, due to protonation/deprotonation processes, they can exist in solution in cationic, anionic and uncharged forms. On the other hand, silica surface can also ionize at changing pH due to the protonation/deprotonation of silanols (Si–OH), as well as incorporated acid-base surface functional groups. Therefore, a change in the pH medium upon formation of the composite can lead to increasing attractive or repulsive drug-silica interactions in the composites. Taking into account the information indicated above, in the present work, the composites of LA with unmodified silica matrix as well as the matrixes modified with different organic groups were synthesized by the sol-gel method at the synthesis pH below or above the pKa of the acid (pKa LA 4.7–5.1 [11]). We focused on the effects of sol-gel synthesis pH and silica matrix modification on the photo and thermal stability of LA in the silica-based composites. LA was used as the racemic mixture of two enantiomers (*R*-(+)-LA and *S*-(−)-LA), because it is presented in the pharmaceuticals and nutraceuticals in most cases.

## 2. Materials and Methods

### 2.1. Reagents

dl-α-Lipoic acid (LA) (>99.0%) (CAS 1077-28-7) was purchased from Tokyo Chemical Industry Co., LTD (Tokyo, Japan). (±) α-Lipoamide (LPA) (96%) (CAS 940-69-2) was purchased from Toronto Research Chemicals (Toronto, ON, Canada). Tetraethoxysilane (TEOS) (ECOS-1, high purity grade, Moscow, Russia); ethanol (EtOH) (95%, Chimmed, analytical grade, Moscow, Russia); (3-mercaptopropyl)trimethoxysilan (MPTMOS) (Sigma-Aldrich, 95%, Geel, Belgium) (CAS 4420-74-0); (3-aminoporopyl)triethoxysilane (APTEOS) (Sigma-Aldrich, 99%, Kenilworth, NJ, USA) (CAS 919-30-2); methyltrimetoxysilane (MTMOS) (Acros, Geel, Belgium) (CAS 1185-55-3); hydrochloric acid (HCl) (Acros, for analysis, 37%, Geel, Belgium) (CAS 7647-01-0) and sodium hydroxide (Chimmed, analytical grade, Moscow, Russia) were used without further purification. Potassium bromide (KBr) (Acros, 99+%, IR grade, Geel, Belgium) (CAS 7758-02-3) was dried at 250 °C before use.

### 2.2. Syntheses of LA Composites with Different Silica Materials

#### 2.2.1. Sol-Gel Synthesis of Composites of LA with Unmodified Silica (UMS) at pH 3 and 7

TEOS (10 g), water (2.16 g), EtOH (6.78 g) and HCl 2M (0.3 mL) were added to flask under stirring. After homogenization, pH of the prepared sol was adjusted to pH 3 or 7 by using 2M NaOH. Then, a solution of LA in EtOH (250 mg in 7.5 mL) was added quickly, check the pH and the mixture stirred vigorously. A gel was formed, and the stirring was stopped after 40 min. The obtained gel was aged and dried at room temperature for 2–3 days.

#### 2.2.2. Sol-Gel Synthesis of Composites of LA with Mercaptopropyl (MPMS), Aminopropyl (APMS) and Methyl (MMS) Modified Silica Materials at pH 3 and 7

The procedure of the synthesis was similar to that described above, but instead of TEOS, its mixture with modifier (MPTMOS or APTEOS or MTMOS) was taken (9.55 g of TEOS and 0.45 g of the modifier). However, at the synthesis of the composites with aminopropyl-modified silica, before adding the solution of LA, pH was adjusted to pH 3 or 7 by using 2 M HCl.

As LA is photosensitive, the synthesis, storage and investigation of the drugs and their composites with the silica materials were carried out in the dark.

The designation of the synthesized composites, their synthesis conditions and amount of loaded drug are presented in Table 1.

#### 2.2.3. Sol-Gel Synthesis of Silica Matrixes (UMS, MMS, MPMS and APMS) at pH 3 and 7

The procedure of synthesis of the matrixes was similar to that described above, but without addition of LA solution.

### 2.3. Photodegradation Experiment and Analysis of Obtained Data

Photodegradation tests were carried using the batch method. The samples were ground in a mortar carefully before the irradiation. Each solid sample of the drug (0.005 g) or the composite (0.08 g) was placed and spread uniformly as a thin film on glass plate. The powders on the glass plates were discrete enough to allow for uniform irradiation. The samples were kept at a distance of 30 cm away from a UV light source (Feron CAB31B lamp, 18W, luminous power 1010 Lm, λ_max_ = 365 nm) in a mirrored chamber. The temperature in the chamber was kept 25 ± 2 °C. After irradiation at discrete time intervals, each sample was suspended in water-ethanol mixture (40%, *v/v*) and kept for 10 h. Then, the sample was centrifuged to determine the drug concentration using a spectrometer SF-2000 (St. Petersburg, Russia) and calibration plots. The photodegradation of the drugs was determined by monitoring the decrease in the intensity (absorbance) of characteristic absorption peak of the drug released from irradiated sample for 10 h. The amount of the degradated drug was calculated relative to the amount of the drug released from unirradiated sample during the same time. The photodegradation kinetics curves were obtained by plotting the percentage of non-degraded drug as function of irradiation time.

The experimental kinetic curves of photodegradation were described using zero-order (1), first-order (2) and second-order (3) kinetic models. The integral forms of equations of the models are:

(1) $$Q_{0}-Q_{t}=-k_{0}t;\;t_{0.5}=\frac{Q_{0}}{2k_{0}}$$

(2) $$\ln(Q_{0}-Q_{t})=k_{1}t;\;t_{0.5}=\frac{\ln2}{k_{1}}$$

(3) $$\frac{1}{Q_{t}}=k_{2}t+\frac{1}{Q_{0}};\;t_{0.5}=\frac{1}{k_{2}Q_{0}}$$

where Q_0_ is the initial amount of the drug in the sample; Q_t_ is the drug amount in the sample at time t; k_0_, k_1_ and k_2_ are the zero-order, first-order and second-order rate constants and t_0.5_ is the half-life for photodegradation.

### 2.4. Thermal Degradation Experiment

To obtain the information on the stability of free and encapsulated LA in the composites, the amount of LA remaining unchanged after thermal treatment as function of heating time was monitored. For this purpose, the samples were ground in a mortar carefully before heating. Each solid sample of the drug (0.005 g) or the composite (0.08 g) was placed and spread uniformly as a thin film on thin metal plate. The powders on the metal plates were discrete enough to allow for uniform heating and kept at 67 °C for a certain time. Then, the measurement procedure was the same as described in the previous paragraph.

### 2.5. Fourier Transform Infrared (FTIR) Spectra

The FTIR spectra were recorded using a VERTEX 80v (Broker Optik, Ettlingen, Germany) spectrometer at room temperature. The spectra were recorded in the range of 4000 to 400 cm^−1^. The samples were examined as KBr disks.

### 2.6. Zeta Potential Measurements of Synthesized Silica Matrixes

The zeta-potentials of the synthesized silica materials in water- alcohol mixtures (40% *v/v*) with pH 3.0 and 7.0 were measured using an analyzer Zetasizer Nano (Malvern Instruments Ltd., Malvern, United Kingdom). The sample powder was dispersed in the mixtures with the indicated pH and sonicated for 5 min before measurement. The pH was adjusted by using HCl and NaOH solutions.

### 2.7. Scanning Electron Microscopy

The scanning electron microscopy (SEM) images were taken with a Hitachi TM4000 Plus model (Hitachi, Ltd, Tokyo, Japan) at 10 kV. The samples were fixed on carbon tape and flattened on the surface.

### 2.8. Statistics

The photo and thermal degradation measurements were repeated 3 times (three independent experiments), and the results are given as mean values ±standard deviations. Of the three kinetic models of photodegradation tested, the model that best fitted with the obtained data was evaluated based on the correlation coefficients.

## 3. Results

### 3.1. FTIR Spectroscopy

**Figure 2:**
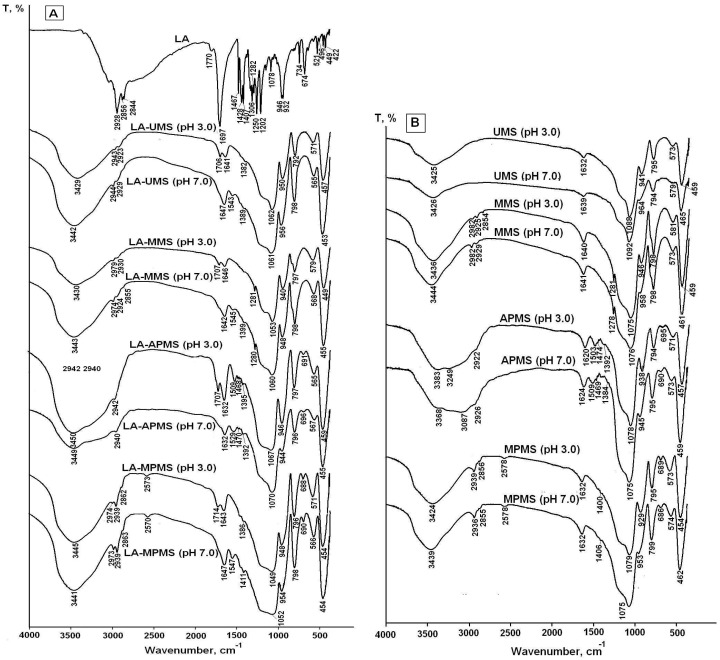
FTIR spectra of crystal LA and LA-silica composites (**A**) and silica matrixes (**B**) synthesized at sol-gel synthesis pH 3 and 7.

As indicated above, drug-silica interactions can play very important role in photo and thermal stabilization of the drug encapsulated in silica. The FTIR spectra are useful tool for investigation of drug-silica interactions [4,5,36,37]. For this purpose, the FTIR spectroscopic investigation of LA interactions with the silica matrixes in the composites was performed. Figure 2 shows the FTIR spectra of crystal LA, the synthesized composites and silica matrixes.

The assignment of FTIR bands in the spectrum of free LA is listed in Table 2. As carboxylic acid, solid lipoic acid exists as H-bonded dimers; therefore, the strong band at 1697 cm^−1^ can be assigned to dimerized lipoic acid. The low intensive band at 1770 cm^−1^ can be assigned to vibrations of free carboxylic groups of the monomer form. The weak ν(S–S) vibrations are found in the 550–400 cm^−1^ region.

The comparison of the spectra of composites with the spectra of corresponding matrixes showed that the spectra of the composites prepared at pH 3 exhibited the bands at 1714–1706 cm^−1^, which can be attributed to vibrations of hydrogen-bonded unionized carboxyl groups of LA molecules [40,43,46]. Due to pKa LA 4.7–5.1 [11], the drug was mainly in uncharged form at sol-gel synthesis pH 3. The carboxyl groups form weak hydrogen bonds with aminopropyl and mercaptopropyl groups of the silica matrixes in LA-APMS (pH 3) and LA-MPMS (pH 3) composites: small shifts of (N–H) deformation vibration and (S–H) stretching vibration bands (+6 cm^−1^ and −5 cm^−1^, respectively) testify about the interaction. Unfortunately, the interactions of LA with the silanol groups (Si–OH) presented in the unmodified and organomodified silica matrixes cannot be clearly detected from the obtained spectra because of overlap of vibrations of the silanols with the stretching O–H vibrations of adsorbed water, LA and N–H bond (in the LA-APMS) in the range of 3700–3200 cm^−1^ [39].

The spectra of the composites prepared at pH 7 exhibited the bands associated with asymmetric carboxylate (COO–) stretching vibrations (1547–1543 cm^−1^) [39,42,43] of LA because at pH 7 the drug existed in anionic form. For LA-APMS (pH 7) and LA-MPMS (pH 7) composites, the shifts of (N–H) deformation vibration band (+20 cm^−1^) and (S–H) stretching vibration band (−8 cm^−1^) were also observed indicating that these groups were involved in interactions with LA. The shift of 20 cm^−1^ for LA-APMS (pH 7) composite may be due to the increased contribution of electrostatic interaction between anionic LA and the positively charged matrix (Table 3).

**Figure 3:**
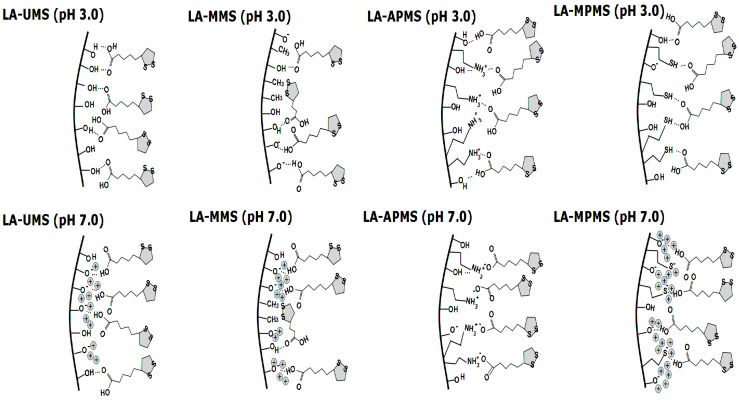
Scheme of possible interactions between LA and the surface of silica matrixes in synthesized.

The interaction in the LA-MPMS (pH 7) composite, as well as probably LA-UMS (pH 7) and LA-MMS (pH 7), occurred contrary to the fact that at pH 7 the surface of all the synthesized matrixes (except APMS) acquired a strong negative charge (Table 3) and LA existed as an anion at the sol-gel synthesis of the composites. Meng et al. [47] studied adsorption of glycine on unmodified silica at different pH and suggested that a local pH near the silica surface may be lower than the value in the solution bulk due to formation of a diffuse layer of opposite charge in the solution neighboring the surface. In this case, a lower local pH may lead to higher percentage of zwitterionic glycine. Possibly, the lower local pH due to diffusion of Na+ ions to the silica surface forming during sol-gel synthesis may promote a higher amount of unionized LA than its bulk value and hydrogen bonding between LA and the silica matrixes in the composites. Thus, in this case, LA located near the silica surface are mainly in unionized form. The bands assigned to S-S bond in 1,2-dithiolane ring of LA molecules are very weak and do not become apparent in the spectra. The dithiolane ring is highly hydrophobic and, possibly, can interact through van der Waals forces with methyl groups of MMS matrix. Figure 3 demonstrates possible interactions occurring at the silica matrix surfaces in the synthesized composites prepared at different pH.

Thus, the FTIR study showed that LA interacts with the silica matrixes in the composites through carbonyl group, and in general, the dithiolane rings of LA molecules do not participate in the interactions.

### 3.2. Study of Photodegradation of LA in the Composites

**Figure 4:**
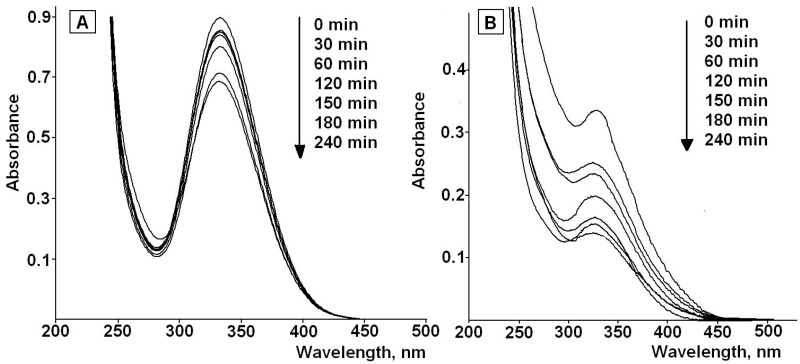
UV-VIS spectra of of LA (**A**) and LA in LA-MPMS composite (**B**) in water-ethanol (40%, *v/v*) during the photodegradation process.

It is well known that the rate of photodegradation and its products depend on the irradiation conditions and, first of all, on the light source [48]. The lamp used at the present work has an irradiation range close to daylight (280–420 nm) with the maximum wavelength at 365 nm. The lamp was used to simulate the radiation of fluorescent lamps at home, during the manufacture process; that is, where the drug can actually undergo photodegradation. The UV-VIS spectra of the solutions of irradiated LA in water-ethanol mixture (40% *v/v*) are presented in Figure 4a. As an example, Figure 4b shows the UV-VIS spectra of the LA in irradiated LA-MPMS (pH 7) in the indicated medium.

The spectra exhibit a decrease in the intensity of the characteristic absorption band of LA at 332 nm with increasing time of the irradiation. This may be due to the rupture of the disulfide bond of 1,2-dithiolane ring in LA molecules and formation of dihydrolipoic acid (DHLA) [14,15,16]. According to literature data, DHLA shows the absorption band at 200 nm [49].

**Figure 5:**
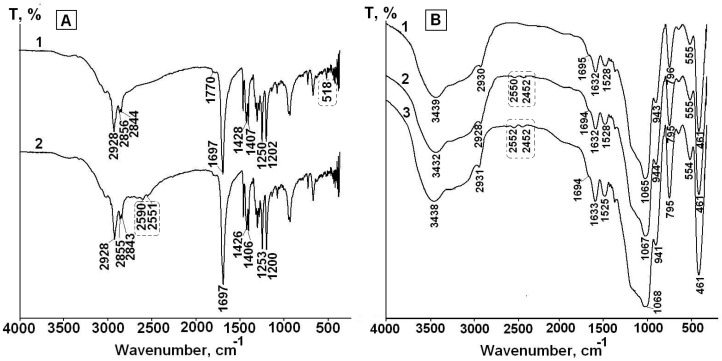
FTIR spectra of solid free LA before (1) and after UV irradiation for 4 h (2) (**A**) and LA in LA-APMS (pH 7) before (1) and after UV irradiation for 2 h (2) and 4 h (3) (**B**).

The FTIR spectra of free LA and the synthesized composites confirm the assumption (Figure 5).

The spectrum of free LA before and after irradiation for 4 h are presented in Figure 5a. The spectrum LA after irradiation (spectrum 2) exhibits the appearance of the bands at 2590 cm^−1^ and 2551 cm^−1^, which are absent in the spectrum of LA before irradiation (spectrum 1) and can be assigned to stretching vibration of S–H bond [39]. Besides, the low intensive band at 518 cm^−1^ presented in the spectrum of unirradiated LA and assigned to stretching vibration of S–S bond [39,45] disappears in spectrum 2.

As an example, the FTIR spectra of LA-APMS (pH 7) composite before and after irradiation for 2 h and 4 h are presented in Figure 5b. Figure 5b shows that in the spectra after irradiation, the bands assigned to stretching vibration of S–H bond (~2600 cm^−1^ and ~2550 cm^−1^) appear. The FTIR spectra of other the synthesized composites exhibited similar feature. Thus, the photodegradation of solid free LA and LA in the composites leads to formation of DHLA.

**Figure 6:**
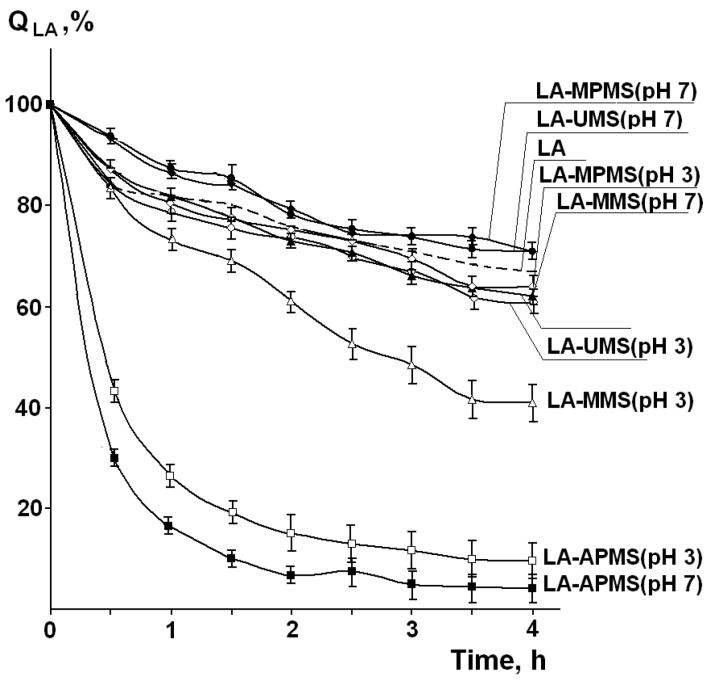
Experimental kinetic curves of photodegradation of free LA and LA in the composites (Q_LA_ is the amount of LA remaining unchanged after the irradiation). (Mean value ± SD, *n* = 3).

In addition to identification of photodegradation products, the important problem in the study of this process is its kinetics, i.e., how quickly drug degrades upon exposure to the light. Figure 6 shows the experimental kinetic curves of photodegradation of free LA and LA in the composites (Q_LA_ is the amount of LA remaining unchanged after the irradiation). It was found that after irradiation of solid LA for 4 h, 66.9% of the drug remained unchanged.

The kinetic curves were fitted with the zero-order, first-order and second-order models, and the obtained kinetic parameters (the rate constants, the half-life for degradation (t_0.5_)) and correlation coefficient values (R^2^) are presented in Table 4.

Regression analysis showed that the photodegradation curves both for free solid LA and LA in most the synthesized composites followed the second-order kinetics (R^2^ = 0.97–0.98). The kinetic parameters for LA-UMS and LA-MPMS composites are close to those for free LA. Among them, LA-UMS (pH 7) and LA-MPMS (pH 7) exhibited a slightly lower rates of photodegradation compared to free LA. The half-life times of LA photodegradation in these composites were the highest (t_0.5_ = 9.1 h and 9.0 h, respectively) and close to that for free LA. For other studied composites, the t_0.5_ values significantly reduced. The higher rate constants for LA-MMS and especially LA-APMS composites indicate that the photodegradation of LA in these composites is significantly more rapid compared to free LA. Thus, the observations demonstrated that the encapsulation of LA in the studied silica matrixes did not prevent photodegradation of LA and even reduced its photostability.

### 3.3. Study of Thermal Degradation of LA in the Composites

LA is unstable under heat. Temperatures greater than its melting point (58–65 °C) cause immediate polymerization of LA [18,19,20,21]. The colorless polymers consist of linear disulfides(II). The formation of colorless polymers results in a decreasing intensity of the absorption band of LA at 332 nm.

**Figure 7:**
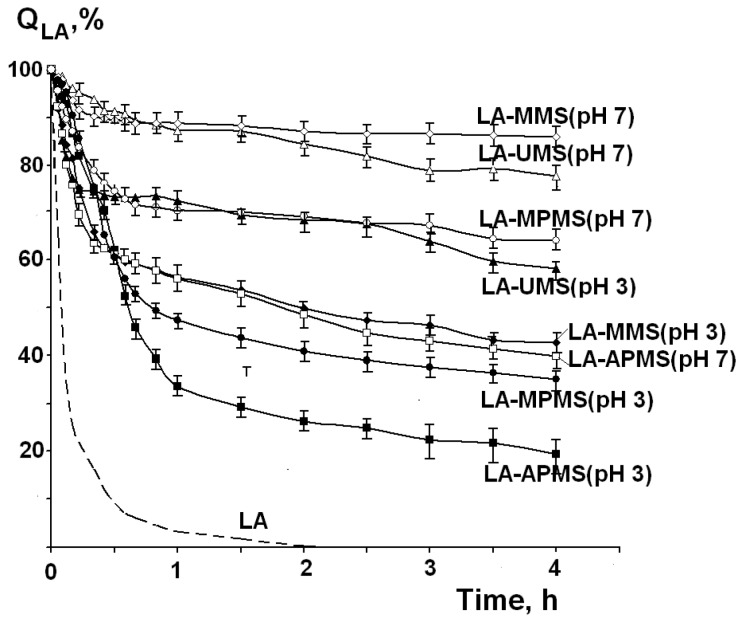
Kinetic curves of thermal degradation of free LA and LA in the composites at temperature 67 °C.

Figure 7 shows the kinetic curves of thermal degradation of free LA and LA in the synthesized composites (Q_LA_ is the amount of LA remaining unchanged after heating). First of all, it should be noted that LA in the composites exhibited a higher thermal stability compared to free LA. As can be seen from the Figure 7, the thermal degradation of LA occurs in two stages: a rapid degradation is observed in the first stage, and then the process slows down. This may be due to degradation of the drug located near the surface of the particles of the composites (the first stage) and then the drug in the depth of the samples (the second stage).

The description of the kinetic curves by the zero-order (1), first-order (2) and second-order (3) models showed a poor correlation of the experimental curves with the applied models. Therefore, the rate constants at the first and the second stages (k_1_ and k_2_) were calculated by linear approximation of the corresponding parts of the curves. The rate constant values, time of the first degradation stage (t_1_) and the amounts of the nondegraded LA (Q_1_, % and Q_2_, %) at the first and second stages are presented in Table 5.

Analysis of the data presented in Table 5 shows that the k_1_ values (i) are higher for the composites prepared at pH 3 compared to the composites prepared at pH 7 and (ii) decrease in the following order for the composites prepared at synthesis pH 3.0:

LA-UMS (pH 3.0) > LA-MMS (pH 3.0) > LA-APMS (pH 3.0) = LA-MPMS (pH 3.0).

The opposite order is observed for the composites prepared at pH 7.0:

LA-APMS (pH 7.0) > LA-MPMS (pH 7.0) > LA-MMS (pH 7.0) > LA-UMS (pH 7.0).

The lowest k_1_ values were observed for LA-UMS (pH 7.0) and LA-MMS (pH 7.0 composites. Among the studied composites, they are the most thermally stable.

## 4. Discussion

**Figure 8:**
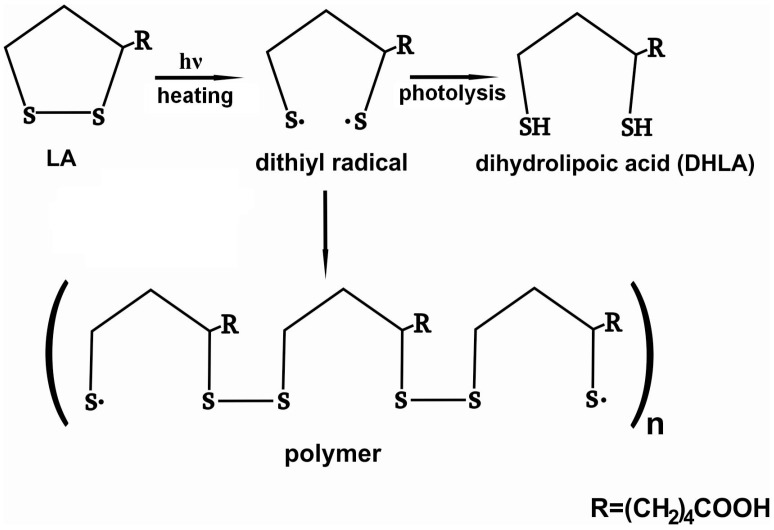
Schematic presentation of products of photo or thermal degradation of LA.

α-lipoic acid molecule contains a disulfide bond in distorted five-membered dithiolane ring, which may be cleaved under UV irradiation or heating. A series of works devoted to investigation of photodegradation of LA has been published by Matsugo et al. [14,15,16]. They suggested that the rupture of the S-S bonds results in formation of dithiyl radicals followed by the intra or intermolecular hydrogen atoms abstraction to form dihydrolipoic acid (DHLA) (Figure 8). The reaction requires the presence of hydrogen to be abstracted by the dithiyl radicals (for example, solvent).

The mechanism was suggested for photodegradation of LA in solution.

In the present work, photodegradation of LA was investigated in the solid LA-silica composites. However, the FTIR spectra (Figure 2a) showed that the composites contained a significant amount of water (the broad bands in the region 4000–3000 cm^−1^ include the stretching vibrations of hydrogen-bonded water molecules; the deformation vibrations of adsorbed water were observed at 1641–1632 cm^−1^ [38,39]), which can be a source of hydrogen atoms for formation of DHLA. The interactions of LA with the silica matrixes cannot impede opening of its dithiolane cycles under UV irradiation and formation of DHLA, since the cycles does not participate in these interactions. Therefore, the mechanism for increasing photostability of the encapsulated LA due to its interaction with the silica matrixes does not work. Besides, Onoue et al. [50] assumed that the lower photostability of coenzyme Q10 in solid emulsified formulations compared to free crystalline Q10 can be explained its amorphous form in the solid formulations. Possibly, the amorphization of LA in the synthesized composites leads to the decrease in its photostability compared to crystalline LA.

**Figure 9:**
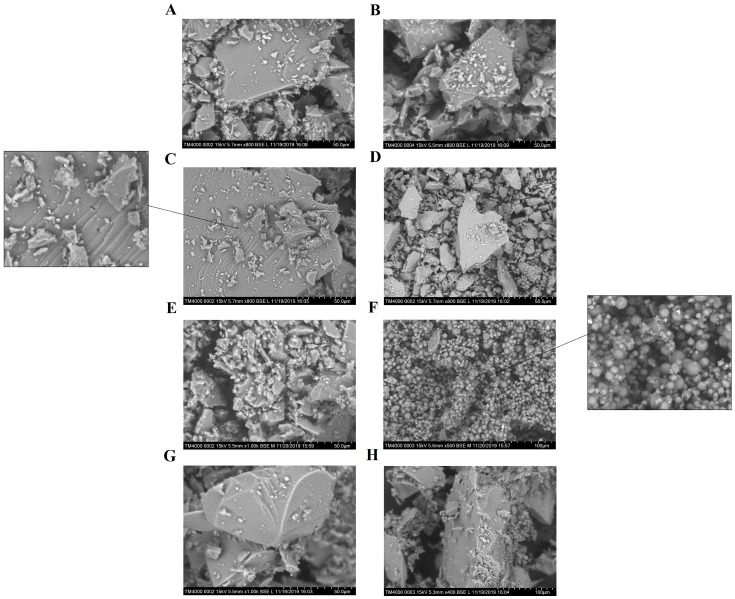
SEM images of the composites: (**A**) LA-UMS (pH 3), (**B**) LA-UMS (pH 7), (**C**) LA-MMS (pH 3), (**D**) LA-MMS (pH 7), (**E**) LA-APMS (pH 3), (**F**) LA-APMS (pH 7), (**G**) LA-MPMS (pH 3) and (**H**) LA-MPMS (pH 7).

Furthermore, it is obvious that most the synthesized matrixes are transparent to UV irradiation. Reducing the effects of the light rays can be a consequence of their reflection from the surface of particles of the composites. Different ability of the materials to reflect irradiation can be associated with different methods of their formation. It is likely that the considerable difference in the photodegradation rates between LA-APMS composites and other composites can be explained by the indicated factor. LA-APMS composites were prepared by precipitation due to strong base nature of (3-aminoporopyl)triethoxysilane (APTEOS). The formation of the remaining composites occurred through a gelation stage, and after aging and drying, they were transparent glasses. This influenced morphology of surface of the composites. Figure 9 shows SEM images of the synthesized composites. LA-UMS, LA-MPMS and LA-MMS composites are composed of smooth grains with quite diverse size and shape, which are aggregates and agglomerates formed due to drying of the composites.

The grains of LA-MMS (pH 3.0) composite have pores of different size. The modification of silica matrix with base aminopropyl groups led to formation of LA-APMS (pH 3) material with some domains of spherical particles. LA-APMS (pH 7) consists entirely of spherical shape particles of various size. The particles are highly porous. It is possible that due to the indicated morphology, the drug in LA-APMS composites is more prone to photodegradation than in the glassy composites, the grain of which can partially reflect the light. The evaluated particle size of LA-APMS composites was 2–7 μm.

As for thermal degradation of LA, according to literature, the process leads to formation of polymer products [18,19,20,21]. To form polymers, LA molecules must lose contacts with the silica matrixes. The stronger the interactions with the matrix, the slower the process of thermal degradation.

In the composites prepared at pH 3, LA in uncharged form interacts with silanol groups of the silica matrixes through C=O···HO hydrogen bonding. The partial substitution of the silanol groups for the organic ones promotes strengthening hydrogen bonding of LA with the organomodified silica matrixes. It is likely that van der Waals interaction of hydrophobic dithiolane rings with CH_3_ groups makes LA-MMS (pH 3) composite stronger.

In the composites prepared at pH 7, the main contribution to thermal stability of the composites gives the hydrogen bonding between the deprotonated silanol groups of the silica matrixes and LA (O^−^···HO), which exists in uncharged form near the silica surface [47]. It is likely that such hydrogen bonding is stronger than O···HO, because the electron-donating ability of the ionized OH group (O^−^) increases strongly [51]. Therefore, the rate constants and the Q_LA_ values are higher for the composites prepared at pH 3.0 compared to those for the composites prepared at pH 7.0. The introduction of the organic groups weakens the interaction in the composites, resulting in increasing rate constants of their degradation.

As has been indicated above, the kinetic curves of thermal degradation of LA in the studied composites clearly exhibited two stages of the process. It may be suggested that the slowdown of the process in the second stage can be associated with the thermal conductivity of the samples, as well as with accumulation of the degradation products.

## 5. Conclusions

The present work is a part of development of novel oral formulation of LA with improved physicochemical and functional properties. It was assumed that encapsulation of the drug in silica matrix would enhance its photo and thermal stability. However, the obtained results were diverse.

We showed that thermal degradation of LA in the synthesized composites proceeded through two stages and was much slower compared to free LA. The cause for this slowdown is the interactions of the drug with the silica matrixes. The stronger the interactions with the matrix, the slower the process of thermal degradation. The highest thermal stability of LA was observed in LA-UMS (pH 7) and LA-MMS (pH 7) composites.

The obtained results on photodegradation of LA in the composites demonstrated that the silica matrixes are not able to protect LA from degradation under UV irradiation despite the LA-silica interactions. Only LA-UMS (pH 7) and LA-MPMS (pH 7) composites exhibited a slightly lower rate of the process compared to free LA. For LA-MMS (pH 3), LA-APMS (pH 3) and LA-APMS (pH 7) composites, a significant acceleration of photodegradation process was observed. This fact was explained by different transparence of the silica matrixes in the composites due to their different surface morphology.

The results reported in this work showed that, from the point of view of photo and thermal stability, the most promising composites for further development of novel silica-based formulations are LA-UMS (pH 7) and LA-MPMS (pH 7), because it was found that LA in these composites degraded under UV light and heated more slowly than free LA.

## Figures and Tables

**Table 1:** Designation of synthesized composites, their synthesis conditions and amount of loaded drug.

Designation of Samples	Precursor	Synthesis pH	Drug Loading, mg/g
LA-UMS (pH 3)	TEOS	3	60
LA-UMS (pH 7)	TEOS	7	57
LA-MPMS (pH 3)	TEOS + MPTMOS	3	59
LA-MPMS (pH 7)	TEOS + MPTMOS	7	58
LA-APMS (pH 3)	TEOS + APTEOS	3	46
LA-APMS (pH 7)	TEOS + APTEOS	7	56
LA-MMS (pH 3)	TEOS + MTMOS	3	60
LA-MMS (pH 7)	TEOS + MTMOS	7	61

**Table 2:** Assignment of FTIR bands in the spectrum of crystal LA.

Band, cm^−1^	Assignment	References
2928, 2856, 2844	asymmetric and symmetric ν (C–H)	[38,39]
1770	ν(C=O)	[39,40,41]
1697	ν(C=O)	[39,42,43]
1467	δ(CH_2_) scissoring	[38,39,43]
1428, 1407		[39,44]
	ν(C–O)/δ(OH) out-of-plane	[42,44]
1306	δ(C–H)	[39,42]
1250	ν(C–O)/δ(OH) out-of-plane	[39,42,43]
1202		[39]
1078		[39,42,43]
946, 932	δ(O–H) out-of-plane	[39,42,45]
734	δ(C–H)	[38,39]
674	ν(C–S)	[39,45]
521, 496, 449	ν(S–S)	[39,45]

**Table 3:** Zeta potentials of synthesized silica matrixes in water-ethanol solutions (40%, *v/v*) with pH 3 and 7.

Zeta-Potentials, mV		
Silica Matrix	pH 3	pH 7
UMS (pH 3)	−0.68	-
UMS (pH 7)	-	−38.0
MMS (pH 3)	−4,78	-
MMS (pH 7)	-	−32.0
APMS (pH 3)	28.9	-
APMS (pH 7)	-	30.8
MPMS (pH 3)	−3.28	-
MPMS (pH 7)	-	−48.5

**Table 4:** Kinetic parameters of photodegradation of free LA and LA in the synthesized composites.

Composite	Zero-Order Model	First-Order Model	Second-Order Model
LA	k_0_ = 7.30 h^−1^; t_0.5_ = 6.9 h R^2^ = 0.8891	k_1_ = 0.09 h^−1^; t_0.5_ = 7.6 h R^2^ = 0.9338	k_2_ = 1.1 × 10**^−3^** h^−1^; t_0.5_ = 9.1 h R^2^ = 0.9793
LA-UMS (pH 3)	k_0_ = 8.30 h^−1^; t_0.5_ = 6.0 h R^2^ = 0.9030	k_1_ = 0.01 h^−1^; t_0.5_ = 6.8 h R^2^ = 0.9393	k_2_ = 1.3 × 10^−3^ h^−1^; t_0.5_ = 7.7 h R^2^ = 0.9844
LA-UMS (pH 7)	k_0_ = 7.45 h^−1^; t_0.5_ = 6.8 h R^2^ = 0.9396	k_1_ = 0.09 h^−1^; t_0.5_ = 7.8 h R^2^ = 0.9554	k_2_ = 0.9 × 10**^−3^** h^−1^; t_0.5_ = 9.1 h R^2^ = 0.9776
LA-MMS (pH 3)	k_0_ = 8.42 h^−1^; t_0.5_ = 5.9 h R^2^ = 0.9153	k_1_ = 0.11 h^−1^; t_0.5_ = 6.3 h R^2^ = 0.9497	k_2_ = 38 × 10^−3^ h^−1^; t_0.5_ = 2.6 h R^2^ = 0.9871
LA-MMS (pH 7)	k_0_ = 14.6 h^−1^; t_0.5_ = 3.4 h R^2^ = 0.8645	k_1_ = 0.23 h^−1^; t_0.5_ = 3.0 h R^2^ = 0.9767	k_2_ = 1.4 × 10^−3^ h^−1^; t_0.5_ = 7.1 h R^2^ = 0.9740
LA-MPMS (pH 3)	k_0_ = 8.25 h^−1^; t_0.5_ = 6.0 h R^2^ = 0.9322	k_1_ = 0.11 h^−1^; t_0.5_ = 3.0 h R^2^ = 0.9767	k_2_ = 1.4 × 10^−3^ h^−1^; t_0.5_ = 7.1 h R^2^ = 0.9730
LA-MPMS (pH 7)	k_0_ = 7.36 h^−1^; t_0.5_ = 6.8 h R2 = 0.9332	k_1_ = 0.09 h^−1^; t_0.5_ = 7.9 h R^2^ = 0.9451	k_2_ = 0.9 × 10^−**3**^ h^−1^; t_0.5_ = 9.0 h R^2^ = 0.9745
LA-APMS (pH 3)	k_0_ = 16.72 h^−1^; t_0.5_ = 2.9 h R^2^ = 0.5972	k_1_ = 0.53 h^−1^; t_0.5_ = 1.3 h R^2^ = 0.8339	k_2_ = 24 × 10^−3^ h^−1^; t_0.5_ = 0.4 h R^2^ = 0.9753
LA-APMS (pH 7)	k_0_ = 15.22 h^−1^; t_0.5_ = 3.3 h R^2^ = 0.4551	k_1_ = 0.67 h^−1^; t_0.5_ = 1.1 h R^2^ = 0.7860	k_2_ = 57 × 10^−3^ h^−1^; t_0.5_ = 0.2 h R^2^ = 0.9751

**Table 5:** Kinetic parameters of degradation LA in composites.

Composite	First Stage				Second Stage			
	k_1_, h^−1^	R^2^	t_1_, h	Q_1_, %	k_2_, h^−1^	R^2^	Q_2_, %	t_2_, h
LA-UMS (pH 3)	133.4	0.9303	0.21	74.9	4.2	0.9690	58.2	3.79
LA-UMS (pH 7)	20.5	0.9708	0.41	91.4	4.0	0.9656	77.5	3.59
LA-MMS (pH 3)	101.1	0.9398	0.41	60.6	5.6	0.9688	42.6	3.59
LA-MMS (pH 7)	33.2	0.9701	0.33	89.4	1.2	0.9441	85.6	3.67
LA-APMS (pH 3)	73.4	0.9730	1.05	33.5	4.4	0.9516	19.3	2.95
LA-APMS (pH 7)	127.6	0.9213	0.32	61.8	6.7	0.9690	39.8	3.68
LA-MPMS (pH 3)	74.2	0.9806	0.67	49.9	4.9	0.9140	35.1	3.33
LA-MPMS (pH 7)	59.3	0.9206	0.50	72.7	2.4	0.9333	64.0	3.5
